# Propofol, midazolam, vancomycin and cyclosporine therapeutic drug monitoring in extracorporeal membrane oxygenation circuits primed with whole human blood

**DOI:** 10.1186/s13054-015-0772-5

**Published:** 2015-02-12

**Authors:** Florian Lemaitre, Nesrine Hasni, Pascal Leprince, Emmanuel Corvol, Ghassen Belhabib, Pierre Fillâtre, Charles-Edouard Luyt, Cyril Leven, Robert Farinotti, Christine Fernandez, Alain Combes

**Affiliations:** Faculty of Pharmacy, EA4123 Barrières physiologiques et réponses thérapeutiques, Paris Sud University, Châtenay-Malabry, France; Department of Clinical and Biological Pharmacology and Pharmacovigilance, Pharmacoepidemiology and Drug Information Center, Rennes University Hospital, Rennes, France; Faculty of Medicine, Laboratory of Experimental and Clinical Pharmacology, Rennes 1 University, Rennes, France; Inserm, CIC-P 1414 Clinical Investigation Center, Rennes, France; Service de Réanimation Médicale, Groupe Hospitalier Pitié-Salpêtrière, iCAN, Institute of Cardiometabolism and Nutrition, 47, Boulevard de l’Hôpital, 75651 Paris, Cedex 13 France; Paris 6 University Pierre et Marie Curie, 91-105 Boulevard de l’Hôpital, 75013 Paris, France; Cardiovascular Surgery department, Pitié-Salpêtrière Hospital, Assistance Publique Hôpitaux de Paris, AP-HP, 47-83 Boulevard de l’Hôpital, 75013 Paris, France; Pharmacy Department, Saint-Antoine Hospital, Assistance Publique Hôpitaux de Paris, AP-HP, 184 rue du faubourg Saint-Antoine, 75012 Paris, France

## Abstract

**Introduction:**

As a result of drug sequestration and increased volume of distribution, the extracorporeal membrane oxygenation (ECMO) procedure might lead to a decrease in drug concentrations during a patient’s treatment. The aim of this study was to evaluate sedative, antibiotic and immunosuppressive drug loss in ECMO circuit using *ex-vivo* and *in-vitro* experiments.

**Methods:**

Blood concentrations of propofol, midazolam, cyclosporine and vancomycin were measured in an *ex-vivo* ECMO circuit primed with whole human blood, and compared to controls stored in polypropylene tubes. *In vitro* experiments were also conducted to further explore the role of temperature, oxygen exposure and polyvinylchloride surfaces on propofol loss in the ECMO circuit.

**Results:**

Propofol concentration decreased rapidly; 70% of its baseline concentration was lost after only 30 minutes, and only 11% remained after five hours (*P* <0.001 for the comparison with control polypropylene tube propofol concentration). Further experiments demonstrated that oxygen exposure and contact with polyvinylchloride tubing were respectively responsible for 70% and 85% of propofol loss after 45 minutes. Midazolam concentration also rapidly decreased in the ECMO circuit, with only 54% and 11% of baseline concentration being detected at 30 minutes and 24 hours respectively (*P* = 0.01 versus control). Alternatively, cyclosporine concentration remained stable for the five first hours, then decreased to 78% and 73% of the baseline value after 24 hours and 48 hours, (*P* = 0.35 versus control). Lastly, vancomycin concentration remained stable in the ECMO circuit for the 48-hour experimental protocol.

**Conclusions:**

We observed important losses of propofol and midazolam, while cyclosporine concentration decreased slowly and moderately, and vancomycin concentration remained unchanged in the *ex-vivo* ECMO circuit primed with whole human blood. These data might help intensive care unit physicians planning clinical trials with a final objective to better adapt doses of these drugs while treating critically ill ECMO patients.

## Introduction

Extracorporeal membrane oxygenation (ECMO) is a complex life-support technique used to rescue critically ill patients with severe respiratory or cardiac failure [1]. Modern ECMO circuits consist of polyvinyl chloride (PVC) tubing, a polymethylpentene membrane oxygenator and a centrifugal pump [2]. Patients on ECMO require multiple medications including sedatives, analgesics, antibiotics and sometimes immunosuppressive drugs [3,4]. Pharmacokinetics of drugs administered during ECMO is complex notably due to a larger volume of distribution in ECMO-treated patients and also to the adsorption of drugs on the PVC tubing and/or the membrane oxygenator leading to an increase in drug clearance [2,5]. The degree of drug uptake by the circuit depends on the physicochemical characteristics of the drugs [6]. For example, compounds with a high octanol/water partition (log P) will be very soluble in organic materials and may exhibit considerable loss in the ECMO circuit [6]. Conversely, the pharmacokinetics of hydrophilic drugs have been reported to be unaffected by the ECMO procedure [7,8]. Drug adsorption on the circuit may also depend on circuit duration of use, since binding sites may become saturated after a few hours of operation [3,9].

To date, very limited data on the pharmacokinetics of drugs in patients supported by ECMO are available. The objective of the present study was, therefore, to determine, in an *ex vivo* ECMO circuit primed with human whole blood, changes in the concentrations of propofol, midazolam, vancomycin and cyclosporine, which are frequently prescribed to ECMO patients.

## Methods

### Drug sequestration in an *ex vivo* ECMO circuit primed with human whole blood

This study was an experimental study and did not include any patients. Thus, it needed no ethical approval and no patient consent was needed. Whole ECMO circuits (Maquet®, Orleans, France) comprising a Quadrox® membrane oxygenator, a centrifugal pump, a heat exchanger and PVC tubing were used for *ex vivo* tests. All components of the circuit were treated with heparin (Bioline coating®, Maquet). Drug-free human whole blood (800 mL) was used to prime the circuit. In order to replicate the *in vivo* operating conditions, the temperature of the circulating blood was set at 37°C and circuit flow rate at 4.5 L/minute. Propofol (Diprivan®10 mg/mL, Fresenius, France), midazolam (Hypnovel 1 mg/mL, Roche, France), vancomycin (Vancomycine® 50 mg/mL, Sandoz, France), and cyclosporine (Sandimmun® 50 mg/mL, Novartis, France) were introduced into the circuit to achieve final concentrations of 2 μg/mL, 30 μg/mL, 500 ng/mL and 1.2 μg/mL, respectively. Octanol/water partition coefficients of the drugs are provided in Table 1. A high partition coefficient is associated with a high solubility in organic materials. Human blood was provided by the Etablissement Français du Sang (EFS) (Rungis, France).

**Table 1:** **Octanol/water partition coefficients of propofol, midazolam, cyclosporine and vancomycin**

**Drug**	**Partition coefficient (log P)**
Vancomycin	- 3.1
Cyclosporine	2.9
Midazolam	3.9
Propofol	4.0

During *ex vivo* ECMO runs, serial post-membrane blood samples were obtained at 30 minutes, 1 hour, 2 hours, 3 hours, 4 hours, 5 hours, 24 hours and 48 hours after drug introduction into the circuit. Until analysis, samples were kept at 4°C and plasma separated and frozen at −20°C. To determine spontaneous drug degradation, blood containing identical concentrations of the drugs studied was kept in polypropylene tubes under agitation at 25°C for the same time before measurements.

Propofol and midazolam concentrations were measured using high performance liquid chromatography with an ultra-violet detector (Waters, Milford, MA, USA). Vancomycin and cyclosporine concentrations were measured using an immuno-enzymatic method on a Dimension® system (Siemens, Munich, Germany).

The operating conditions (oxygen flow, blood flow, circuit temperature) remained stable during the 48 hours of each experiment which were repeated three times with three different ECMO circuits.

### Impact of temperature, oxygen and polyvinylchloride surfaces on propofol concentrations *in vitro*

To further explore the role of temperature, oxygen exposure and PVC surfaces on propofol loss in the ECMO circuit, the following experiments were conducted. A polypropylene tube containing human whole blood was spiked with the propofol stock solution to obtain a concentration of 2 μg/L. The effect of the temperature, oxygen exposure and contact with PVC surfaces were assessed by incubating the tube at 37°C, applying oxygen (1 L/minute) for 10 seconds to the preparation and by distributing the preparation into 5-cm PVC tubes, respectively. Polypropylene tubes containing blood and propofol at the same concentration were stored at room temperature and were used as controls. Propofol concentrations were measured at 0 minutes, 5 minutes, 10 minutes, 15 minutes, 20 minutes, 25 minutes, 30 minutes and 45 minutes from each preparation. These experiments were conducted in triplicate.

### Statistical analyses

Changes of mean drug concentrations over time were compared between control and experimental conditions by modeling using a linear mixed effect model. This model accounts for the repeated responses from the same experiments by using a random effect on intercept solely (propofol and vancomycin) and using a random effects on intercept and time (midazolam and cyclosporine). Model choice was guided by the lowest Akaike criteria value. The mixed effect model was then fitted with R software version 3.0.2 and the ‘nlme’ library [10].

## Results

### Changes in drug concentrations in *ex vivo* circuits primed with whole human blood

**Figure 1:**
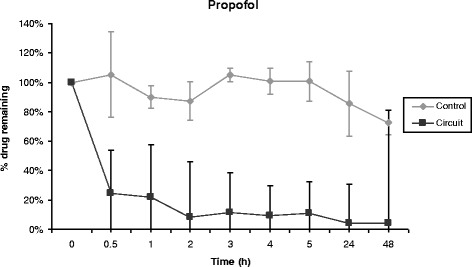
**Percentage of propofol remaining in the ECMO circuit according to time (mean ± standard error of the mean; n = 3).** ECMO, extracorporeal membrane oxygenation.

**Figure 2:**
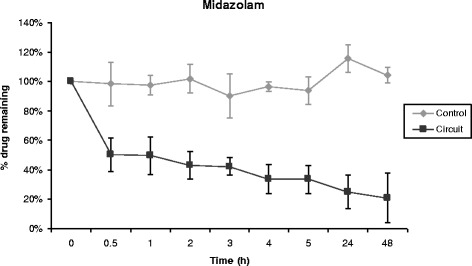
**Percentage of midazolam remaining in the ECMO circuit according to time (mean ± standard error of the mean; n = 3).** ECMO, extracorporeal membrane oxygenation.

**Figure 3:**
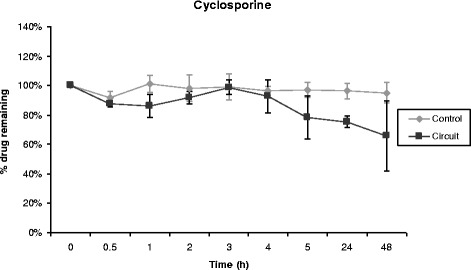
**Percentage of cyclosporine remaining in the ECMO circuit according to time (mean ± standard error of the mean; n = 3).** ECMO, extracorporeal membrane oxygenation.

**Figure 4:**
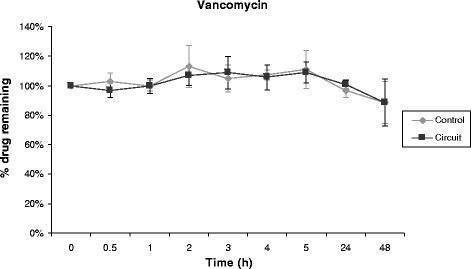
**Percentage of vancomycin remaining in the ECMO circuit according to time (mean ± standard error of the mean; n = 3).** ECMO, extracorporeal membrane oxygenation.

Propofol, midazolam, cyclosporine and vancomycin concentrations remained stable over time in control polypropylene tubes. Alternatively, propofol concentration decreased rapidly in the ECMO circuit, 70% of its baseline concentration being lost only 30 minutes after introduction of the drug into the circuit (Figure 1). After 5 hours, only 11% of the initial propofol concentration remained and the fraction remaining beyond 24 hours was negligible. Differences between experiment and control polypropylene tube propofol concentrations were statistically significant (*P* = 0.0006). Midazolam concentration also decreased rapidly in ECMO circuits (Figure 2), with only 54% and 11% of baseline concentrations being detected at 30 minutes and 24 hours, respectively (*P* = 0.01 versus polypropylene control). The cyclosporine concentration remained stable in the circuit for the first 5 hours, then decreased to 78% and 73% of the baseline value after 24 hours and 48 hours, respectively (*P* = 0.35 versus polypropylene control) (Figure 3). In contrast, vancomycin concentration remained stable in the ECMO circuit during the 48 hours of the experimental protocol (*P* = 0.86) (Figure 4).

### Impact of temperature, oxygen and PVC surfaces on propofol concentration *in vitro*

**Figure 5:**
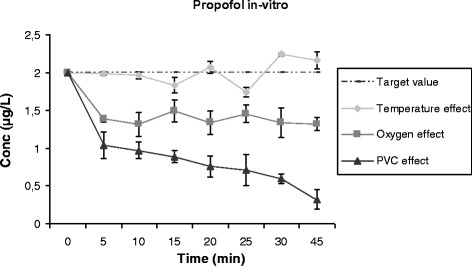
**Effect of temperature, oxygen and PVC contact on propofol concentrations (mean ± standard error of the mean; n = 3).** PVC, polyvinyl chloride.

Oxygen exposure and contact with PVC tubing resulted in 70% and 85% loss of propofol after 45 minutes, respectively. Alternatively, heating the polypropylene tube to 37°C had no effect on propofol concentration (Figure 5).

## Discussion

This study showed important losses of propofol and midazolam in *ex vivo* ECMO circuits primed with whole human blood, while cyclosporine concentration decreased slowly and moderately and vancomycin concentration remained unchanged. Furthermore, we demonstrated that oxygen exposure and contact with PVC tubing were major factors leading to propofol degradation in ECMO circuits.

Patients on ECMO frequently receive many drugs, such as sedatives and analgesics, antibiotics or immunosuppressants. Inadequate blood concentration of these drugs might lead to treatment failure or toxicity, underlining the need for better knowledge of the complex pharmacokinetics and pharmacodynamics in this particular setting [11,12]. Indeed, drug concentrations in critically ill ECMO patients might be altered by an increase in drug volume of distribution or alteration in drug elimination. Drugs might also be adsorbed in the ECMO circuit, with greater loss being reported for more lipophilic and higher octanol/water partition coefficient drugs (Table 1) [6,13-16].

Consistent with previous reports [1,17], we observed a rapid and major decrease in propofol concentration in our experiments. Although its lipophilic properties might be associated with substantial loss due to adsorption on circuit components, our results suggested that oxidation might also be an important determinant of propofol degradation. Indeed, propofol is a reducing agent which can be oxidized in the presence of an electron acceptor [18].

The decrease in midazolam concentration was rapid and only 25% of the initial concentration remained in the circuit after 24 hours. This observation is also consistent with previously reported *in vitro* and *ex vivo* data on midazolam concentration evolution in ECMO circuits, with the decrease reaching 68% to 87% after 24 hours [1,6,19].

Inadequate concentrations of antibacterial agents can lead to treatment failure that might compromise the outcomes of critically ill patients. Vancomycin, which is the drug of choice for treating beta-lactam resistant Gram-positive bacteria, is frequently prescribed to ECMO patients. Data on vancomycin concentrations reported in ECMO are controversial. In agreement with our observation, Mehta *et al.* [20] and Shekar *et al*. [19], using *ex vivo* models, found that vancomycin levels remained unchanged in the circuit for 24 hours. Conversely, Dagan *et al.* observed that vancomycin concentrations decreased by 36% when the drug was introduced in a new circuit while it decreased by only 11% when introduced in a circuit which had been running for five days [3]. Saturation of adsorption sites was suggested by these authors [3]. Similarly, Wildschut *et al.* also reported a decrease in vancomycin concentration (up to 46%) in a study conducted with neonatal and pediatric circuits [6]. These observations suggest that more data are needed to better determine changes in drug concentrations of vancomycin in an ECMO circuit.

Immunosuppressive agents, such as cyclosporine, are defined by a narrow therapeutic window and low serum concentrations of these drugs might lead to acute graft rejection. Although a previous study suggested *in vitro* adsorption studies of cyclosporine on PVC infusion sets significantly higher than that in those made of polyethylene or polybutadiene [21], this study is the first to evaluate cyclosporine pharmacokinetics in ECMO patients. Our results suggested that cyclosporine concentration decreases slowly in the ECMO circuit with 66% of the initial concentration remaining in the circuit after 48 hours. This decrease might be attributed in part to adsorption on the ECMO circuit component, given its high partition coefficient (log P = 2.92).

## Conclusions

We reported herein on propofol, midazolam, vancomycin and cyclosporine concentration profiles in *ex vivo* whole-blood primed ECMO circuits. Our observations showed important losses of propofol and midazolam, while cyclosporine concentration decreased slowly and moderately and vancomycin concentration remained unchanged. These data might help intensive care unit physicians better adapt doses of these drugs while treating critically ill ECMO patients. Further studies should focus on the *in vivo* pharmacokinetics of drugs during ECMO.

## Key messages

- This paper confirmed important loss of lipophilic drugs within ECMO circuits.
- It also highlighted the role of oxidation as an influencing factor of *ex vivo* stability of reducing drugs.
- Drug properties should be taken into account for therapeutic strategy in ECMO patients.

## Abbreviations

ECMO: Extracorporeal membrane oxygenation
EFS: Etablissement Français du Sang
PVC: Polyvinyl chloride
